# A New Method to Protect Blood Supply in the Treatment of Femoral Neck Fractures: Bidirectional Compression Porous Tantalum Screws

**DOI:** 10.1111/os.13285

**Published:** 2022-07-18

**Authors:** Dewei Zhao, Zhijie Ma, Baoyi Liu, Lei Yang, Xing Qiu, Simiao Tian, Chukwuemeka Samuel Okoye, Zhiqiang Lian

**Affiliations:** ^1^ Department of Orthopedics Affiliated Zhongshan Hospital of Dalian University Dalian China; ^2^ The Orthopedic Research Centre, Affiliated Zhongshan Hospital of Dalian University Dalian China; ^3^ Department of Engineering Mechanics State Key Laboratory of Structural Analysis for Industrial Equipment, Dalian University of Technology Dalian China

**Keywords:** central fixation, femoral neck fracture, porous tantalum screws, protect blood supply, postoperative complications

## Abstract

**Objective:**

To explore the clinical effect of a new type of bidirectional pressurized porous tantalum screw (PTS) internal fixation in treating femoral neck fractures (FNFs).

**Methods:**

In this study, geometric models of FNF were first established via reverse engineering method, followed by stimulation of the strength of PTSs in fixation of FNFs. A randomized control trial study was then conducted of 41 patients with FNF from October 2015 to December 2018. These patients included 12 males and 29 females with an average age of 59.9. The 41 patients were randomly divided into two groups: cannulated compression screws (CCSs) group (*n* = 21) and PTSs group (*n* = 20). Treatment outcomes in patients were evaluated using multiple imaging techniques, including X‐ray and digital subtraction angiography scanning as well as functional recovery Harris hip score. Without other postoperative complications, the primary outcome was defined as fracture healing after FNF internal fixation. Secondary outcomes are the incidence of the avascular necrosis of femoral head (ANFH), fracture nonunion, and reoperation rate.

**Results:**

Following PTS internal fixation of FNF, finite element results revealed a firmly fixed fracture with a slight displacement of less than 0.5 mm. At follow‐up, we found a statistically significant difference in Harris scores in the two groups at 1 month and 3 months post‐surgery. In the PTSs group, there was no case of ANFH and fracture nonunion, and the average healing time was 94.45 ± 6.47 days. In the CCSs group, there were four cases of ANFH, the necrosis rate was 19.05% (4/21). There was one case of fracture nonunion in the CCSs group, the nonunion fracture rate was 4.76% (1/21), and the average healing time was 122.54 ± 11.37 days. Five patients underwent total hip arthroplasty, and the reoperation rate was 23.81% (5/21). There were significant differences in the postoperative complications, fracture healing time, and reoperation rate between the two groups (*p* < 0.05).

**Conclusions:**

PTSs fixation of FNF at the center, does not only avoid the destruction of blood supply in the femoral head and reduction in the incidence of postoperative complications of FNFs, but also induces early bone ingrowth and promotes fracture healing. These findings provide a potential surgical internal fixation system for treating FNFs.

## Graphical abstract

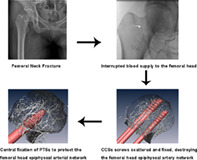

Cannulated compression screws internal fixation for femoral neck fractures can destroy the blood supply of the femoral head. The central fixation with tantalum metal screws can protect the femoral head epiphyseal arterial network and reduce postoperative complications.

## Introduction

Femoral neck fractures (FNFs) are commonly encountered injuries in clinical practice, accounting for about 54% of hip fractures^1^. Once FNF is diagnosed, it should be surgically treated in time to restore blood circulation to the femoral head as early as possible to promote fracture healing^2^. Although the application of internal fixation technology has increased the healing rate of FNFs to more than 90%^3^, which has greatly improved the prognosis of patients, the nonunion rate of FNFs after internal fixation is as high as 10% to 34%, while the necrosis rate is as high as 35% to 48%^4^, ^5^, ^6^. Nonunion of FNFs and avascular necrosis of femoral head (ANFH) require reoperation, even total hip arthroplasty, resulting in tremendous psychological and economic burden to patients^7^. With the advancement of knowledge in anatomy, physiology, and biomechanics of FNFs, clinicians have designed many methods to ensure fracture healing and reduce the incidence of avascular necrosis of the femoral head, by using partial osteotomy which converts the shear force at the end of the fracture into pressure, facilitating fracture healing, and reconstruction of the blood supply of the fracture using bone grafts with muscle, blood vessel pedicles, or free bone grafts^8^, ^9^. However, these methods do not mitigate the destruction of the femoral head epiphyseal arterial network caused by screw implantation and these methods are associated with relatively large trauma and different clinical effect, which limits their clinical application^10^, ^11^. The treatment of FNF still remains a complex problem.

The currently recognized etiologic theory of traumatic ANFH shows that a displaced FNF may result in rupture, distortion, and compression of the blood vessels supplying blood to the femoral head, resulting to ANFH^12^, ^13^. The viability of the femoral head after FNF depends on the preservation of the rest of the vascular supply, as well as the revascularization and repair of the necrotic areas before the necrotic bone segment collapses. Therefore, early accurate reduction, stable internal fixation, preserving the rest of the blood supply to the femoral head, and promoting blood perfusion are essential factors in avoiding postoperative complications^2^. Zhao *et al*.^14^ evaluated the residual biood supply of femoral heads in 27 patients with femoral neck fractures before surgery by analyzing digital subtraction angiography (DSA) data. Preliminary research by our team shows that the epiphyseal arterial network and the inferior retinacular arterial system are two significant structures that maintain femoral head blood supply after FNFs. However, most commonly used implants for the treatment of FNFs are cannulated compression screws (CCSs)^9^, ^15^. During the fixation of fracture, multiple and discrete implantations of these screws will inevitably destroy the arterial network in the epiphysis of the femoral head, further aggravating the injury of the femoral neck blood circulation and ultimately increasing the risk of ANFH and fracture nonunion^16^.

Based on these studies, the researchers analyzed the residual blood supply in the femoral head after FNF and the related factors of the implant's damage to the blood supply and generated the characteristics of a new implant specially designed to fix FNF. This new implant was named “Bidirectional Pressurized Porous Tantalum Screws.” This clinical trial aims to clarify these two questions: (i) Is the clinical treatment effect of porous tantalum screws (PTSs) better than CCSs in the treatment of FNFs? (ii) Can PTSs central fixation effectively protect the residual blood supply of the femoral head after FNFs, and reduce the postoperative complications and reoperation rate after FNFs internal fixation?

## Methods

### Design Features of PTSs

**Fig. 1:**
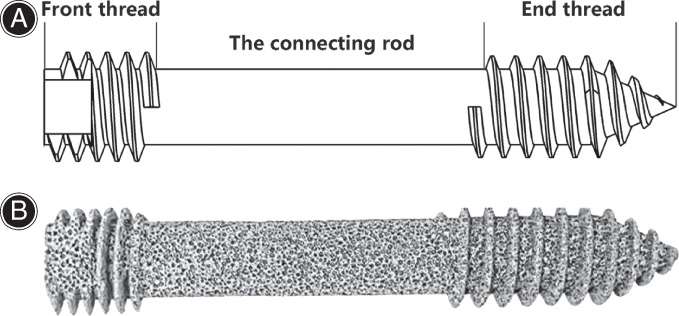
Bidirectional compression porous tantalum screws: (A) Schematic diagram of screws; (B) Porous tantalum screw image

Porous tantalum metal was prepared by our team using a previously reported method^17^, which was then machined into PTSs using a spark‐forming machine. PTS design features: There are threads at the front and rear ends, the thread height is 2 mm, the front end thread pitch is 3 mm, the tail end thread pitch is 2.5 mm, the shaft is 10 mm in diameter, and the screw length is 70–110 mm. The bidirectional compression screw has controlled axial compression. The tail of the screw body was designed with flat bulges to ensure the screw body is easily implanted into the femoral neck and firmly fixed the FNF (Figure 1).

### Inclusion and Exclusion Standards for Patients with FNFs

The inclusion criteria were: (i) Garden's classification^18^ I to III type FNF; (ii) ages 18–80 years; (iii) unilateral fracture; (iv) complete clinical data and written informed consent from the patient.

The exclusion criteria were: (i) multiple or compound injuries; (ii) Garden IV type FNF; (iii) pathological FNF; (iv) diabetes mellitus or vascular disease affecting the lower extremities.

### Material and Group of Patients

**Fig. 2:**
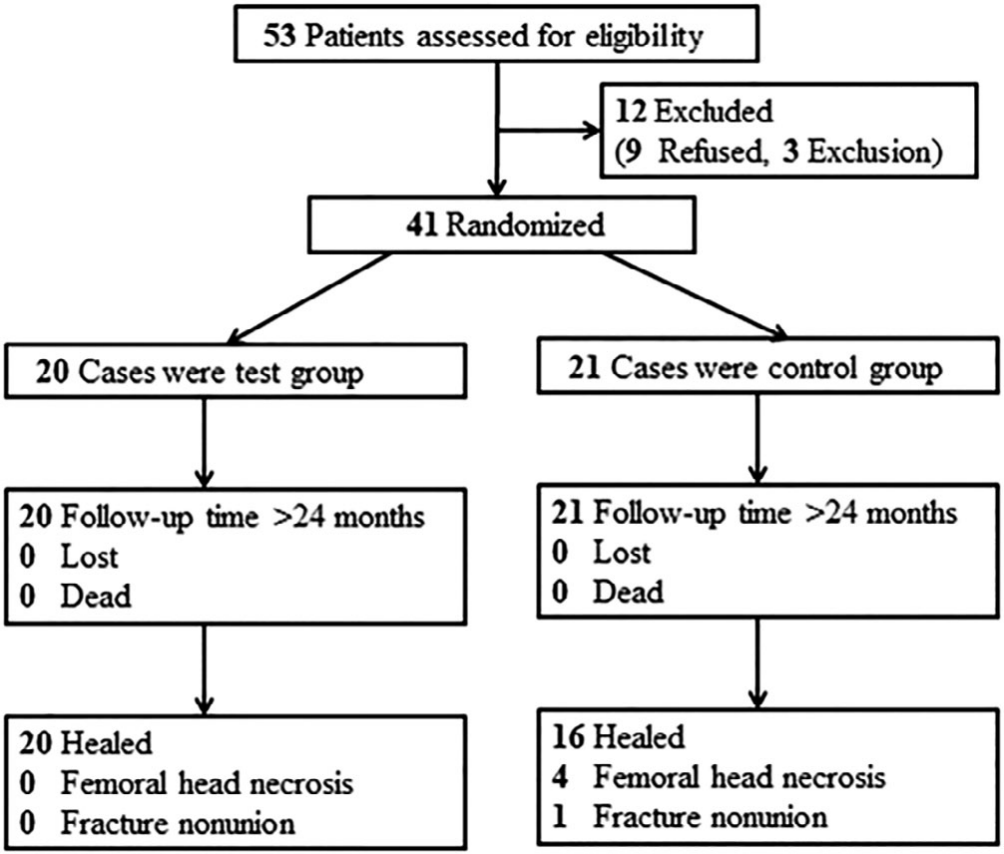
CONSORT (Consolidated Standards of Reporting Trials) flowchart

From October 1, 2015 to December 30, 2018, 41 patients (12 male and 29 female) aged 33–78 years (average age: 59.9 years) with FNFs were qualified and recruited for the current study. Patients meeting the inclusion criteria were randomly divided into two groups: the PTSs group and the CCSs group. All injuries were considered closed fractures. Figure 2 shows the Consolidated Standards of Reporting Trials flowchart.

### Finite Element Model and Calculations

In this paper, the authors used reverse engineering method to create the geometrical model of FNF. The complete flowchart of modeling is outlined as follows: (i) segment the computed tomography (CT) images by a certain threshold value to get the outline of femoral head and femoral neck; (ii) export the outline of the femoral head and femoral neck; (iii) translate the outline curves to point clouds. Here, some reverse‐engineering techniques were used to construct the geometrical models, such as sample, smooth, relax, wrap, detect and edit contours, construct mesh, fit surface, 3D compare, etc. Finally, the geometric models in NURBS form were established and saved as IGES file format.

To establish the subject‐specific finite element models, the CT of a 65‐year‐old patient with FNF, Garden II, was used as a subject. Fast CT (Sensation 64; Siemens) with 2 mm slice interval and 512 × 512 resolution was used to scan the femur of the patient applying the 120 KV voltage. One hundred and fifty pictures were obtained and saved in DICOM format. The element type of the model was set as solid 187, the elastic modulus of screw was 4.8 GPa, and the Poisson's ratio was 0.3. The boundary condition was fixed for all translations at the bottom of femur, and it was assumed that the model is in the normal stand position of the body, with an external load R of 1725 N and M of 1266 N (the body is 75 kg for clinical case). A 10 mm× 80 mm screw model was established by using NX 10.0 software (GRE, Siemens Inc.). Then, the model was imported into ANSYS software for finite element analysis and fractured femur head.

### Operational Procedure

**Fig. 3:**
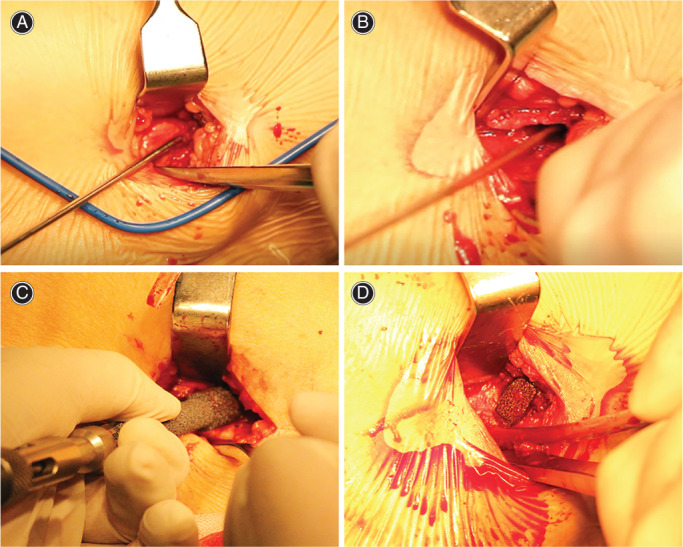
Surgical flow chart: (A) After closed reduction, determine the needle entry point. (B) Drilling. (C) Implant screws. (D) The fracture was firmly fixed

The patient is laced supine on a fracture table, and the Whitman technique was used for closed reduction. The placement of the inferocentral wire was localized using fluoroscopy in both planes. Make a 2–3 cm surgical incision at the distal end of the greater trochanter, separate the vastus lateralis muscle and expose the proximal femur. CCSs group: Three annulated compression screws (7.3 mm) in an inverted triangle configuration were used to fix the FNF. The inferocentral wire was entirely placed on both views. Appropriate anteversion can be determined with the help of putting a guidewire along the anterior femoral neck. After the position of the first guide pin was satisfied, the posterosuperior and anterosuperior pins were used to get posterior and anterior cortical support in the femoral neck by a parallel guide. The threaded guide pins were advanced near the articular surface without violating the articular surface. The length of the guide pin was measured and 5 mm was subtracted to determine the appropriate screw length. Washers were allowed if space permits. PTSs group: PTSs with a diameter of 10 mm was used to fix FNFs. The inferocentral wire was perfectly placed on both views. A guide pin was placed along the center of the femoral neck for fixation. The threaded guide pins were advanced near the articular surface without violating the articular surface. Measure the length of the guide pin and subtract 10 mm to determine the appropriate screw length. The surgical procedure is shown in Figure 3. Patients received these surgeries by an experienced surgeon.

### Assessment of the Treatment Effects

#### Hip Joint Function Evaluation

Hip joint function was accessed using the Harris hip score (HHS)^19^. This study evaluated the hip joint function of the patients before surgery and at 1‐, 3‐, and 6‐months postoperative time. HHS is an objective index for evaluating hip joint function. The standard uses a 100‐point scoring system. The evaluation content includes pain, walking function, activities of daily living, extent of deformity, and joint range of motion, with higher values indicating greater functionality. Clinical outcomes were categorized using HHS in this manner: excellent, 90–100; good, 80–89; fair, 70–79; poor, less than 70.

#### Radiological Assessment

Anteroposterior radiographs were assessed for fracture healing and complications. The follow‐up was conducted at 6 weeks, 3 months, 12 months, and 24 months. According to the definition, the initial result index, failure of fixation, means the requirement for repair surgery due to nonunion, ANFH, or implant cut‐out.

#### Evaluating Blood Supply by DSA Examination

DSA evaluated the femoral head blood supply among the patients with FNF before and after operation.

This study assessed the extent of damage of the superior and inferior retinacular arteries that supply blood to the femoral head using DSA. The authors considered the vessel “uninterrupted” when the contrast agent passes through the retinacular vessels, and eventually lead to the femoral head. Otherwise, the vessel was considered “interrupted.”

### Statistical Analysis

The features of the research population was presented as the mean and SD or median (interquartile range) for continuous variables, or as numbers and percentages for categorical variables. Shapiro–Wilk test was first used to determine whether the age variable followed a normal distribution and it has been shown as non‐significant. The results of two groups were compared using Student's *t*‐test for age, Mann–Whitney test for follow‐up time, and chi‐square test or Fisher's exact test for categorical variables where appropriate. All analyses were carried out using SPSS 20.0 statistics software, and *p* < 0.05 was considered statistically significant.

## Results

### General Results

The CCSs group and PTSs group showed no significant difference in patient characteristics, including age (*t* = 0.296, *p* = 0.769), sex (*χ* ^2^ < 0.001, *p* = 1), fracture type (*p* = 0.308), and follow‐up time (*W* = 175.5, *p* = 0.375) (Table 1). There were no adverse events during the care of these patients.

**TABLE 1:** General characteristics of patients

	All participants (*n* = 41)	PTSs Group (*n* = 20)	CCSs Group (*n* = 21)	*t*/*χ* ^2^/*W* value	*p* value^a^
Age	59.95 (10.38)	60.45 (11.33)	59.48 (9.64)	0.296	0.769
Sex				<0.001	1
Men	10 (24.39%)	5 (25.00%)	5 (23.81%)		
Women	31 (75.61%)	15 (75.00%)	16 (76.19%)		
Type				‐^b^	0.308
I	3 (7.32%)	3 (15.00%)	0 (0.00%)		
II	15 (36.59%)	7 (35.00%)	8 (38.10%)		
III	23 (56.10%)	10 (50.00%)	13 (61.90%)		
Follow‐up time	36.0 (31–47)	34.5 (26.5–49.0)	38.0 (33.0–42.0)	175.5	0.375

Abbreviations: CCS, cannulated compression screw; PTS, porous tantalum screw

^a^ There was no significant difference in patient characteristics between the CCSs group and PTSs group

^b^ For Fisher's exact test, only *p*‐value was provided for 2 × 3 contingency Table.

### Finite Element Analysis

**Fig. 4:**
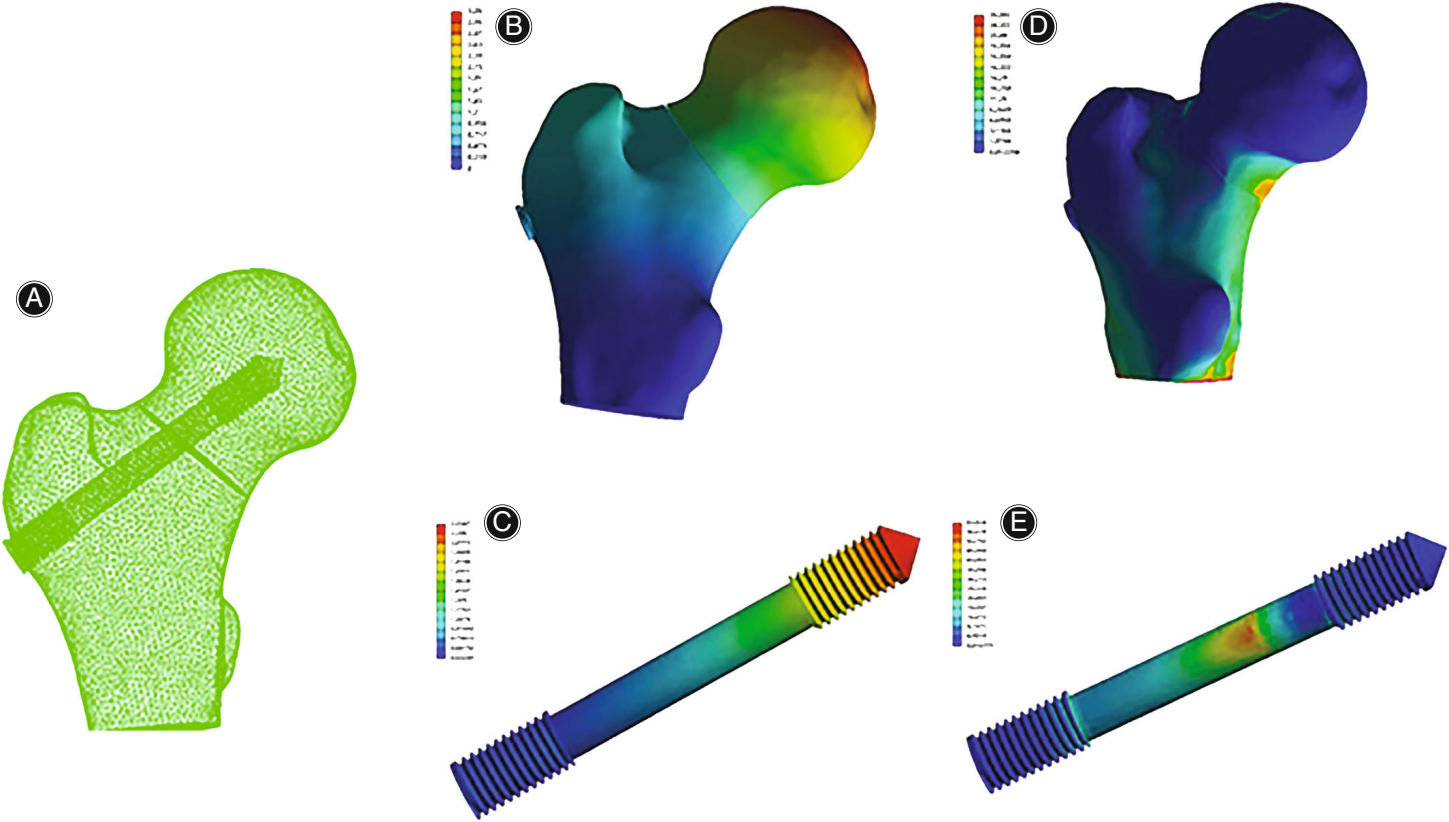
Finite element model analysis of bidirectional compression porous tantalum screw for fixation of femoral neck fractures. (A) Schematic diagram of the method of fixation of femoral neck fractures with PTS screws; (B, C) As can be seen from the displacement contours, the maximum displacement was 3.06 mm, which occurred at the top of the femoral head and screw. However, there is a relatively slight displacement at the femoral neck fracture, and the relative displacement is less than 0.5 mm. (D, E) It can be seen from the stress nephogram that the maximum stress occurs at the constraint position of the lower end of the femur, and the maximum Von Mises stress is 26.58 MPa. PTS, porous tantalum screw

With ANSYS finite element analysis software, the schematic diagram of PTS screw fixation of FNF is shown in Figure 4A; from the displacement contours, it is shown that the maximum displacement was 3.06 mm, occurring at the top of the femoral head and screw. There is a relatively slight displacement at the FNF, and the relative displacement is less than 0.5 mm (Figure 4B,C). The maximum Von Mises stress is 26.58 MPa, occurring at the constraint position at the lower end of the femur (Figure 4D,E).

### Clinical Examinations

The operation time, intraoperative blood loss, and hospitalization length were compared between the two groups. Independent sample *t*‐test shows no significant difference in operation time (*t* = 0.685, *p* = 0.497), intraoperative blood loss (*t* = 0.678, *p* = 0.862), and hospitalization length (*t* = 0.611, *p* = 0.333) between the two groups (Table 2). Two groups showed a statistically significant difference in Harris scores at 1 month (*t* = 35.24, *p* < 0.0001) and 3 months (*t* = 20.14, *p* < 0.0001) after surgery. There was no significant difference at 6 months (*t* = 0.923, *p* = 0.351) after surgery (Table 3).

**TABLE 2:** Comparisons of surgical outcomes in the two groups

Group	Operation time (min)	Blood loss (ml)	Hospitalization length (days)
PTSs	64.40 ± 4.29	147.30 ± 32.63	12.25 ± 1.37
CCSs	63.38 ± 5.17	140.50 ± 31.38	11.95 ± 1.72
*t* values	0.685	0.678	0.611
*p* values	0.497	0.862	0.333

Abbreviations: CCS, cannulated compression screw; PTS, porous tantalum screw.

**TABLE 3:** Harris hip score (HHS) after femoral neck fracture internal fixation

Group	Post. 1 month	Post. 3 months	Post. 6 months
PTSs	68.55 ± 1.17	79.06 ± 1.13	88.04 ± 1.54
CCSs	52.09 ± 1.12	69.91 ± 1.09	86.85 ± 3.91
*t* values	35.240	20.140	0.923
*p* values^a^	<0.0001	<0.0001	0.351

Abbreviations: CCS, cannulated compression screw; PTS, porous tantalum screw

^a^ Harris scores between the two groups at 1 month, 3 months, and 6 months after surgery.

### Digital Subtraction Angiography Examination

**Fig. 5:**
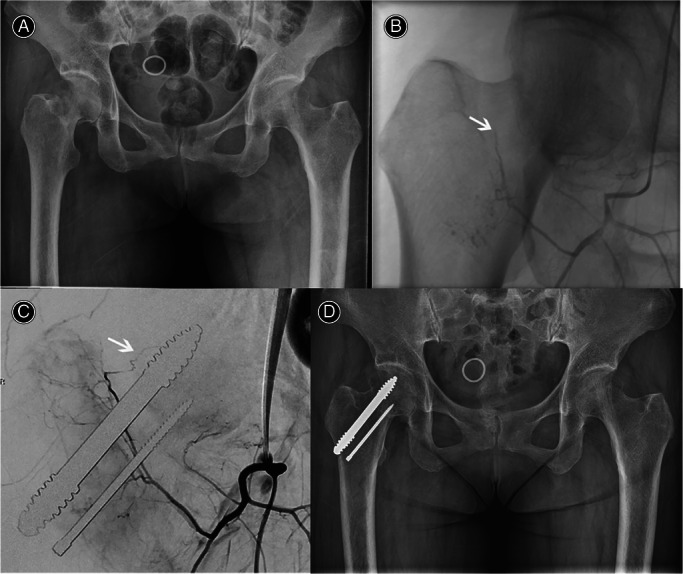
(A) A 78‐year‐old female, FNF (Garden III) of right. (B) Preoperative DSA examination showed that the blood supply of the inferior retinacula arterial existed. The superior retinacula arterial supplying blood to the femoral head was distorted and compressed, and the blood supply was interrupted. (C) The results of DSA at 24 months after surgery showed that the blood supply of the superior retinacula arterial and the inferior retinacula arterial exists, without damaging the epiphyseal arterial network of the femoral head. The white arrow indicates the superior retinal artery of the femoral head, and the red arrow indicates the inferior retinal artery. (D) Twenty‐four months post‐operation, the fracture healed, PTS screw was firmly fixed, and no complications of ANFH occurred. ANFH, avascular necrosis of femoral head; FNF, femoral neck fracture; PTS, porous tantalum screw

**Fig. 6:**
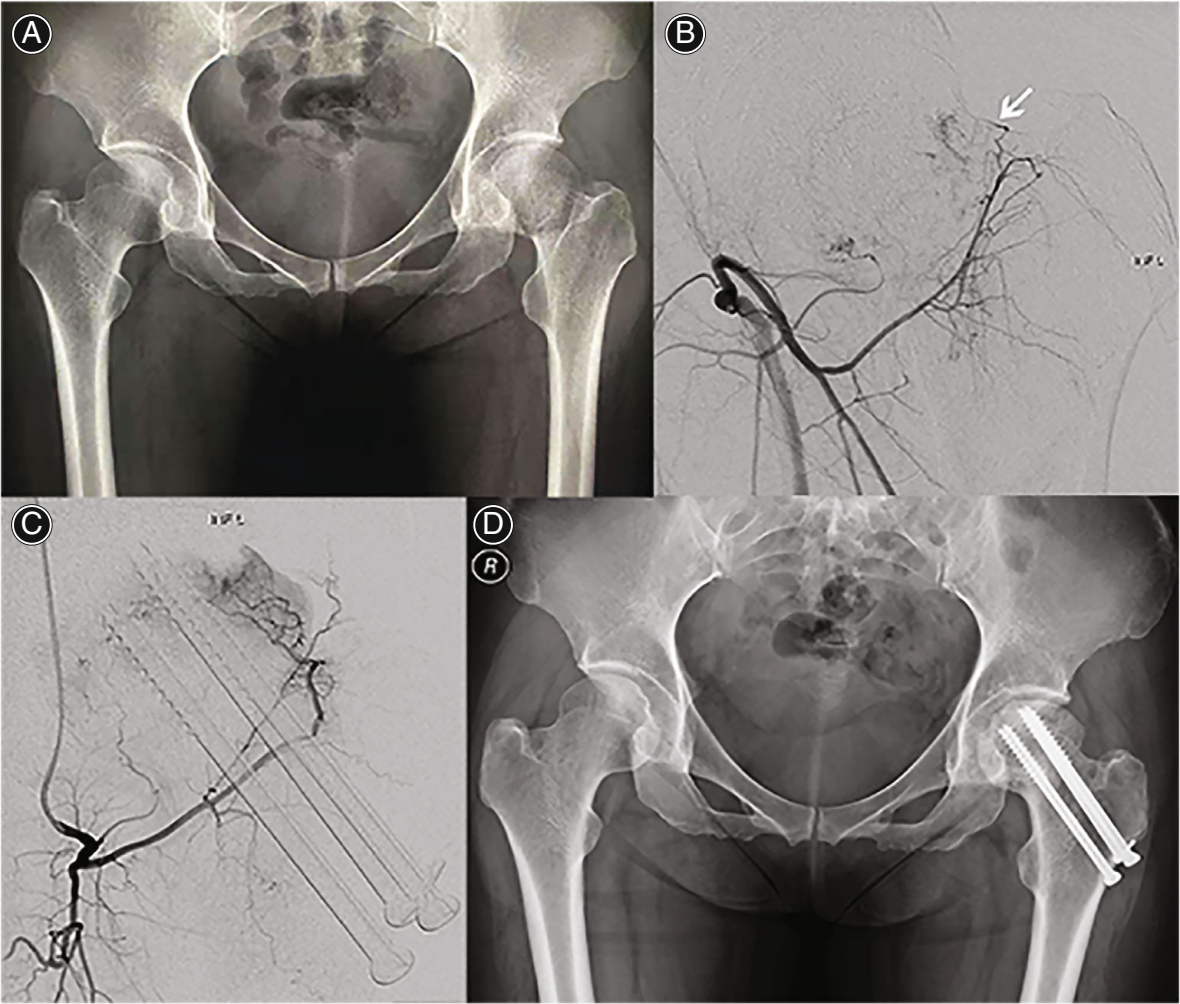
(A) A 35‐year‐old female, FNF (Garden II) of left. (B) Preoperative DSA examination showed that the blood supply of the superior retinacular arterial and the inferior retinacula arterial exists. (C) The results of DSA at 24 months after surgery showed that the superior retinacula arterial and the inferior retinacula arterial blood supply were interrupted. (D) Twenty‐four months after internal fixation with CCSs, avascular necrosis of the left femoral head occurred with collapse of the femoral head, and the screws penetrated the femoral head. CCS, cannulated compression screw; FNF, femoral neck fracture

The DSA results of Garden type I to III FNF patients showed that the superior and inferior retinacular arteries supplying blood to the femoral head persist after the fracture (Figures 5 and 6). Twenty‐four months after surgery, the blood supply of the superior retinacula arterial and the inferior retinacula arterial existed in the PTSs group, without damaging the epiphyseal arterial network of the femoral head (Figure 5C), and there was no ANFH and fracture nonunion. Figure 6C shows that the superior retinacula arterial and inferior retinacula blood supply were interrupted in the CCSs group. The main reason is that the three CCSs were dispersed fixed and multiple implanted, which destroyed the femoral head epiphyseal artery network and eventually led to ANFH.

### Radiological Assessment and Postoperative Complications

Through continuous radiological evaluation, the FNFs in the PTSs group were all healed completely. There were no complications of ANFH and fracture nonunion, and the average healing time was 94.45 ± 6.47 days. The average fracture healing time in the CCSs group was 122.54 ± 11.37 days. During the follow‐up period, four patients from the CSSs group developed hip pain at 14, 17, 18, and 18 months after the operation. Imaging examination showed necrosis and collapse of the femoral head, and the screw passed through the femoral head. The incidence of femoral head necrosis was 19.05% (4/21). One patient had fracture nonunion 11 months after operation, the screw loosened, and the patient moved with crutches, and the fracture nonunion rate was 4.76% (1/21). After complications occurred, all five patients received total hip arthroplasty, and all of them worked and lived normally after the operation. The reoperation rate was 23.81% (5/21).

## Discussion

The viability of the femoral head after FNF depends on preserving the rest of the vascular supply and the revascularization and repair of the necrotic areas before the necrotic bone segment collapses. When using CCSs to treat FNFs, the three screws were scattered and fixed, the (re)vascularization of the femoral head was further impaired, probably because the incidence of vascular necrosis is increased. These features suggest that the central area of the femoral head should be safer than the peripheral region while drilling and screw placement (damages to the main stems of epiphyseal arteries and the lateral region of the epiphyseal arterial network shall be avoided). Therefore, this research prepared PTSs to achieve this purpose: while firmly fixing the FNF at the center and protecting the blood supply of the femoral head, it can promote fracture healing, achieve long‐term biological fixation, reduce or even avoid the series of complications after FNFs with promising biological and mechanical properties.

### Clinical Effect of PTSs for Treating Femoral Neck

This study applied two different internal fixation methods to treat 41 cases of FNFs. PTSs groups and CCSs groups showed a statistically variation in Harris scores at 1 month and 3 months after surgery, respectively (Table 3). The main reason for this difference is the axial compression and rough porous structure of the PTSs, which increasing the screw's grip on cancellous bone with a rotational stability. PTSs do not only prevent screw rotation, but also fix the fracture firmly. After the operation, the patient can move immediately with the help of crutches, and the fractured site will not be displaced. Early exercise can reduce or eliminate long‐term complications of being bedridden. Two groups showed no statistical difference in hip scores with the prolongation of follow‐up time, i.e. 6 months after surgery; because these patients undergoing surgery can walk normally, the difference in hip function scores between the two groups was gradually reduced.

During the follow‐up period, the average healing time of FNF in the PTSs group and CCSs group was 94.45 ± 6.47 days and 122.54 ± 11.37 days, respectively. The two groups showed significant variations in the fracture healing time (*p* < 0.05). The fracture healing time of the PTSs group was shortened, which is mainly related to the good osseointegration property of porous tantalum metal, which is consistent with previous clinical studies^20^, ^21^, ^22^.

### PTSs Center Fixation Can Effectively Protect the Blood Supply of the Femoral Head

The postoperative complication rate in the CCSs group was 23.81%, consistent with previous studies^10^, ^12^. The causes of fracture nonunion and ANFH are mainly associated with the blood supply in the femoral head. As two important structures, the epiphyseal arterial network and the inferior retinacular arterial systems maintain femoral head blood supply after FNF. According to the “high residual rate” feature of the inferior retinacular artery, the femoral head blood supply maintenance after the injury is made through the inferior retinacular arteries‐inferior epiphyseal arteries‐epiphyseal arterial network and inferior retinacular arteries‐metaphyseal arterial network‐epiphyseal arterial network pathways, exerting a significantly compensatory effect after FNF. In this study, DSA was performed on patients with complications of ANFH after treating FNFs with hollow compression screws. The DSA results showed that the superior retinacula arterial and the inferior retinacula arterial blood supply were interrupted. Three screws were dispersing fixed, which destroyed the femoral head epiphyseal artery network, and eventually led to ANFH. Without postoperative complications in the PTSs groups, the main reason was central fixation, which did not destroy the epiphyseal artery network within the femoral head. These results are consistent with study by Zhao *et al*.^16^.

These results showed that the epiphyseal arterial network could be a significant structure for maintaining the femoral head blood supply after FNFs. Through increasing efforts to protect the femoral head epiphyseal arterial network during surgery, the role of iatrogenic injury of intraosseous vascular system might be reduced, preventing fracture nonunion and ANFH.

### Limitations

This study has few limitations. The sample size of the clinical control study is relatively small, which needs to be further confirmed in multi‐centers and by using a large‐sample control study.

### Conclusion

In this study, PTSs have been designed specifically for treating FNFs. They were characterized by combining dynamic compression, center fixation, and rotational stability, which avoids the destruction of blood supply within the femoral head. Preclinical experiment shows that PTSs decrease the incidence of FNFs postoperative complications, induce early bone ingrowth, and promote fracture healing. This study provides a potential surgical internal fixation system for treating FNFs.
